# NGF signaling in PC12 cells: the cooperation of p75^NTR^ with TrkA is needed for the activation of both mTORC2 and the PI3K signalling cascade

**DOI:** 10.1242/bio.20135116

**Published:** 2013-07-12

**Authors:** Sara Negrini, Rosalba D'Alessandro, Jacopo Meldolesi

**Affiliations:** 1 San Raffaele Scientific Institute, DIBIT, via Olgettina 58, 20132, Milan, Italy; 2 Vita-Salute San Raffaele University, Division of Neuroscience, via Olgettina 58, 20132 Milan, Italy; 3 S. De Bellis Scientific Institute, Castellana Grotte, 70013 Bari, Italy

**Keywords:** REST, PC12-27, ERK and PI3K cascades, mTORC2, mTORC1-dependent feed-back inhibition

## Abstract

PC12-27, a PC12 clone characterized by high levels of the transcription repressor REST and by very low mTORC2 activity, had been shown to be unresponsive to NGF, possibly because of its lack of the specific TrkA receptor. The neurotrophin receptor repressed by high REST in PC12-27 cells, however, is shown now to be not TrkA, which is normal, but p75^NTR^, whose expression is inhibited at the transcriptional level. When treated with NGF, the PC12-27 cells lacking p75^NTR^ exhibited a defective TrkA autophosphorylation restricted, however, to the TrkA(Y490) site, and an impairment of the PI3K signaling cascade. This defect was sustained in part by a mTORC1-dependent feed-back inhibition that in wtPC12 cells appeared marginal. Transfection of p75^NTR^ to a level and surface distribution analogous to wtPC12 did not modify various high REST-dependent properties of PC12-27 cells such as high β-catenin, low TSC2 and high proliferation rate. In contrast, the defective PI3K signaling cascade and its associated mTORC2 activity were largely rescued together with the NGF-induced neurite outgrowth response. These changes were not due to p75^NTR^ alone but required its cooperation with TrkA. Our results demonstrate that, in PC12, high REST induces alterations of NGF signaling which, however, are indirect, dependent on the repression of p75^NTR^; and that the well-known potentiation by p75^NTR^ of the TrkA signaling does not concern all the effects induced by NGF but primarily the PI3K cascade and its associated mTORC2, a complex known to play an important role in neural cell differentiation.

## Introduction

p75^NTR^, a receptor of the tumor necrosis factor receptor superfamily, is known to induce different effects depending on its interacting partners. Together with a trans-membrane protein, sortilin, p75^NTR^ activated by the pro-neurotrophins induces apoptotic responses; together with LINGO-1 and Nogo-A participates in the myelin-dependent inhibition of axonal growth (Bandtlow and Dechant, 2004; Barker, 2004; Nykjaer et al., 2005); together with the neurotrophin tyrosine kinase receptors, the Trks, promotes survival, axonal growth and differentiation of neural cells (Bandtlow and Dechant, 2004; Barker, 2004; Nykjaer et al., 2005; Reichardt, 2006). The latter effects, investigated primarily in the pheochromocytoma PC12 cell model treated with NGF (Greene and Tischler, 1976), were reported to depend on the increased affinity (Barker and Murphy, 1992; Hempstead et al., 1991) and the potentiated signaling (Barker, 2004; Reichardt, 2006) of TrkA induced by its interaction with p75^NTR^. Numerous mechanisms were proposed to account for the effects of p75^NTR^: increased ceramide signaling (Brann et al., 1999) and increased L1CAM expression (Itoh et al., 1995); activation of the PI3K signaling cascade (Roux et al., 2001); reduced ubiquitination of TrkA (Makkerh et al., 2005) accompanied by its increased endocytosis and retrograde transport (Geetha et al., 2005); activation of the NF-kB and Sall2 transcription factors with ensuing changes of gene expression (Pincheira et al., 2009); secretase-induced cleavage of p75^NTR^ itself, with release of its intracellular domain, first to the cell cytoplasm and then possibly to the nucleus (Ceni et al., 2010; Kanning et al., 2003; Parkhurst et al., 2010). Whatever the mechanism, the cooperation of p75^NTR^ with TrkA is now recognized to be of great importance. Without p75^NTR^ the response to NGF of the TrkA-expressing cells is reduced, in some cases strongly (Bui et al., 2002; Ceni et al., 2010; Zhang et al., 2012).

Part of the results summarized so far were obtained by employing PC12 cells complemented in their NGF receptors (see, for example, Ceni et al., 2010; Ito et al., 2003; Zhang et al., 2012). Other studies were carried out using PC12 clones spontaneously different from the wild type PC12 (wtPC12), for example with the PC12nnr5 clone that lacks the TrkA receptor (Loeb et al., 1991) and the PC12D clone characterized by its rapid NGF-induced neurite sprouting (Sano and Iwanaga, 1996). In another clone, PC12-27, isolated in our laboratory, the response to NGF was greatly defective but was rescued by the over-expression of exogenous TrkA (Leoni et al., 1999). Based on these findings the defective NGF-induced response of PC12-27 cells was attributed to their lack of the TrkA receptor (Leoni et al., 1999). At the moment, however, direct evidence of this lack is based only on immunocytochemistry (Schulte et al., 2010).

In our previous studies, many differences of PC12-27 with respect to wtPC12 cells were shown to depend on their high level of the transcription repressor REST (RE-1 Silencer of Transcription, also known as NRSF), a master factor of neural cell specificity (Ballas and Mandel, 2005; Ooi and Wood, 2007). Indeed, the level of REST in PC12-27 exceeds by 60–70-fold the very low level typical of neurons and neural cells, including wtPC12 (D'Alessandro et al., 2008). The consequences of the high REST of PC12-27 cells are multiple. On the one hand, many neural cell-specific proteins encoded by REST target genes, such as those of regulated neurosecretion, are lacking (D'Alessandro et al., 2008); on the other hand, PC12-27 cells exhibit accelerated cell proliferation, controlled by a signaling loop in which REST operates together with the GAP protein TSC2 and the co-transcription factor β-catenin (Tomasoni et al., 2011). The activities of the two mTOR complexes were found to be dissociated: that of mTORC1 was slightly higher, that of mTORC2 was very low (Tomasoni et al., 2011), much lower than those of wtPC12, of other neural cells and of various types of neurons (Carson et al., 2013; Mazei-Robison et al., 2011; Urbanska et al., 2012). Whether and to what extent these properties of PC12-27 cells depend on their defective NGF signaling had never been established.

Here we have employed the high REST PC12-27 cells as a model to investigate the possible role of REST in the signaling of the two types of NGF receptors, TrkA and p75^NTR^. Our aim was to obtain new information about the role of the two receptors, and of their cooperation, in the expression of properties typical of the PC12 cell line. We have found that PC12-27 cells lack not the TrkA, as previously hypothesized (Leoni et al., 1999; Schulte et al., 2010), but the p75^NTR^ receptor. This lack induces in the cells a defect of NGF signaling which, however, is not general but affects especially the PI3K cascade, the activity of mTORC2 and the neurite outgrowth. Our results identify new aspects of the cooperation of TrkA and p75^NTR^, of great relevance for neural cell function and differentiation.

## Results

### NGF receptor expression and signaling

**Fig. 1.**
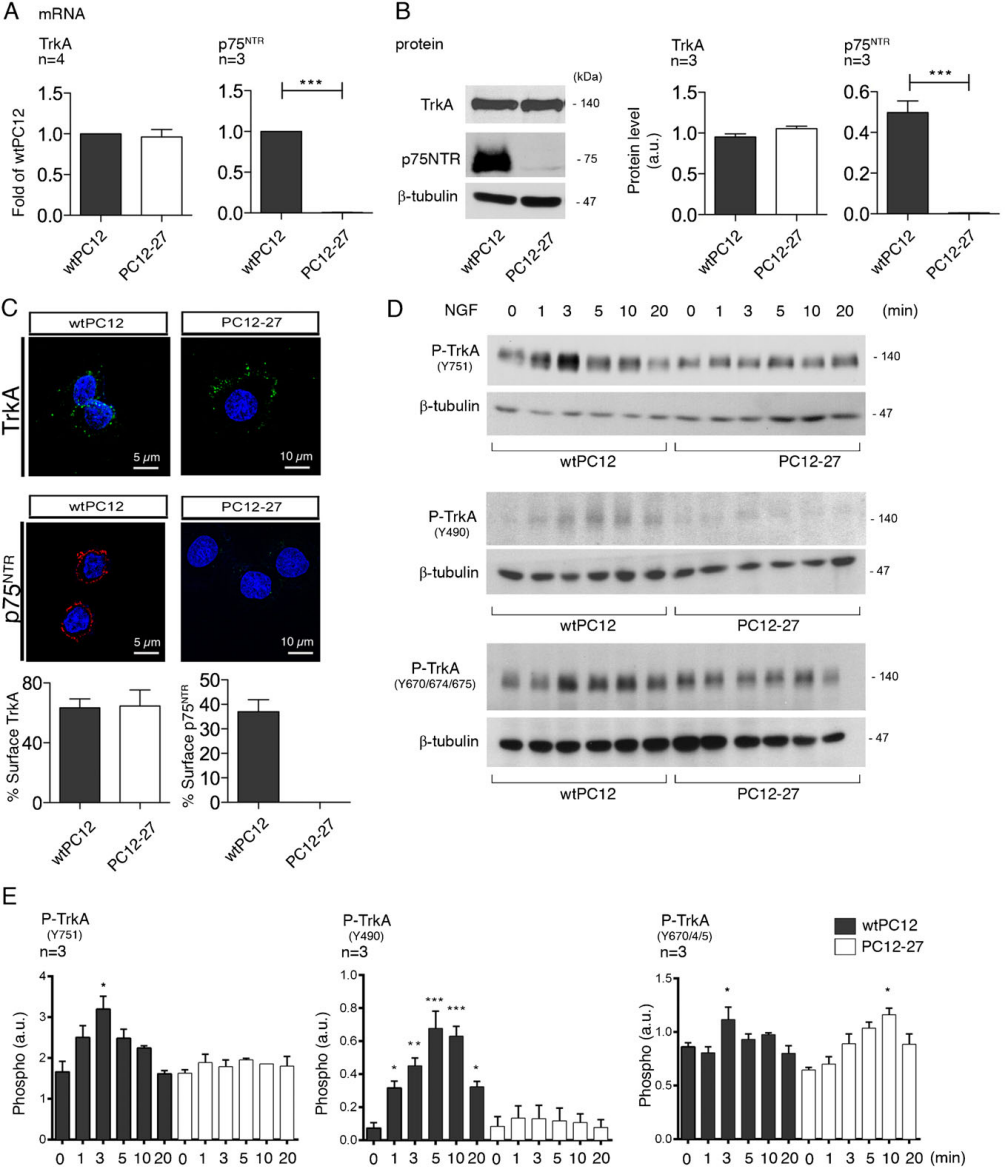
Expression of TrkA and p75^NTR^, and NGF-induced TrkA autophosphorylation responses in wtPC12 and PC12-27 cells. (**A**,**B**) The mRNA and protein of the two receptors revealed in the two clones by RT-PCR (A) and western blotting (B). Notice the lack of p75^NTR^ in the PC12-27 clone. (**C**) The surface immunolabeling of the two receptors in the wtPC12 and PC12-27 cells. The fractions of the total receptors distributed to the surface, given as percentages, are shown in the two subpanels below. Scale bars: 5 µm (left), 10 µm (right). (**D**) The time-course of the autophosphorylation induced by NGF (100 ng/ml) at three sites of the TrkA receptor, Y751 on the top, Y490 in the middle and Y670, Y674 and Y675, analyzed together, at the bottom. The time-course data of panel D are shown in quantitative terms in panel **E**. Here, and in the following figures, the number of gels analyzed quantitatively is given by the numbers written over the panels; the numbers flanking the gels are the MDa of the immunolabeled proteins, given only in the figure showing the protein for the first time. The significance of the results, given as averages ± s.e., is calculated with respect to the sample labeled 0 in each cell population. *P<0.05; **P<0.01; ***P<0.001.

In the initial work, the expression and functioning of the NGF receptors, TrkA and p75^NTR^, was compared in two PC12 clones, the wtPC12 and the high REST PC12-27. The levels of the receptors in the two clones, analyzed under resting conditions (10% serum in the medium), are shown in Fig. 1A–C. In contrast to the previous hypothesis of Leoni et al. (Leoni et al., 1999), both the mRNA and the protein of TrkA were close in the two clones. In contrast p75^NTR^, which is prominent in wtPC12, was inappreciable in PC12-27 cells at both the mRNA and the protein level (Fig. 1A,B). These results could be due to a direct repression of p75^NTR^ by the high REST of PC12-27 cells. In fact the gene of p75^NTR^ includes in its promoter two copies of RE-1, the DNA sequence specific of REST binding. In contrast, no RE-1 sequence is present in the promoter of TrkA (Wu and Xie, 2006; Bruce et al., 2004). When immunolabeled with antibodies specific for TrkA and p75^NTR^ (Fig. 1C), the wtPC12 cells were positive both before and after detergent permeabilization, two conditions that reveal the surface and total complement of the receptors, respectively. In the PC12-27 cells the TrkA immunolabeling was very similar to that of wtPC12, whereas the p75^NTR^ was completely negative (Fig. 1C). In terms of localization, the TrkA, expressed by the cells of the two clones was mostly exposed at the cell surface while the p75^NTR^ of wtPC12 was ∼40% surface-exposed and ∼60% retained within the cells (Fig. 1C).

We next investigated the effects of NGF on its receptor signaling. During preliminary studies we investigated the ERK 1 and 2 (ERK 1/2) and Akt phosphorylation effects induced in the wtPC12 and PC12-27 clones by various concentrations of NGF: 2, 25 and 100 ng/ml. At the lower concentrations the effects induced by the neurotrophin were inappreciable or small. Only with the highest concentration many of the differences induced by NGF reached the level of significance. In view of these preliminary results, the subsequent studies were most often carried out using NGF at 100 ng/ml, a concentration widely employed in recent studies (see, among others, Koch et al., 2008; Miranda et al., 2001; Pincheira et al., 2009; Wang et al., 2013).

**Fig. 2.**
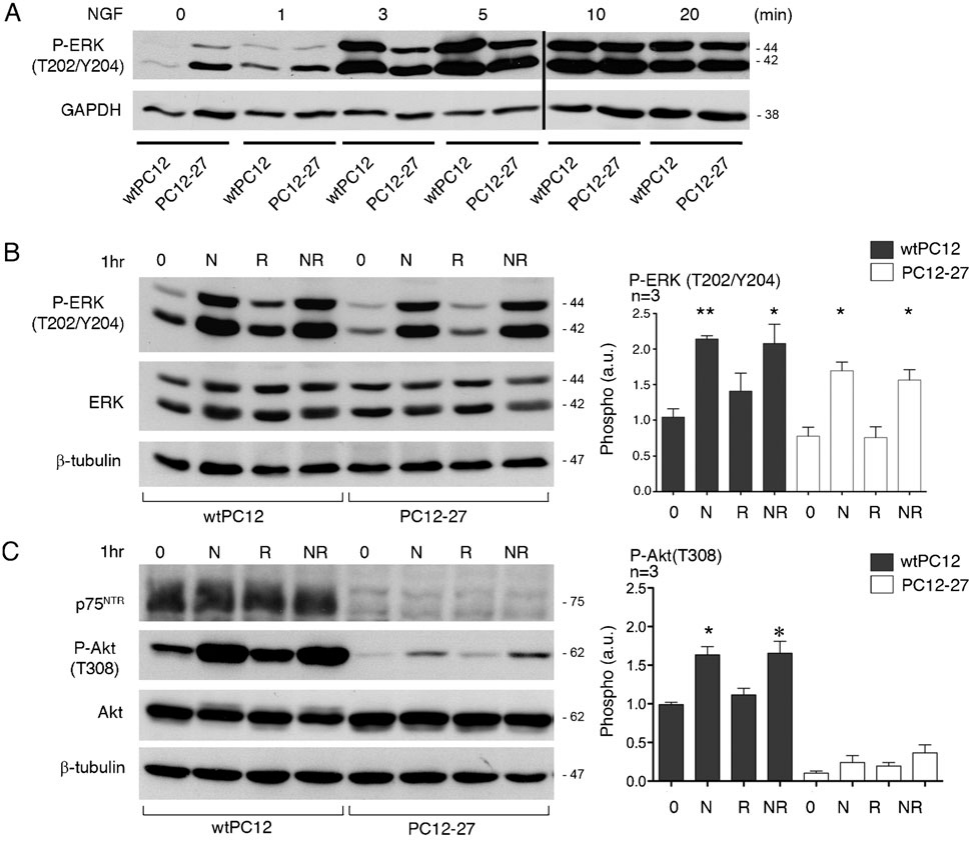
ERK and PI3K signaling cascades in wtPC12 and PC12-27 cells. (**A**) The time-course of the ERK 1/2 phosphorylation induced by NGF (100 ng/ml) at the T202/Y204 sites analyzed together. (**B**) The phosphorylation responses induced at the T202/Y204 sites of ERK 1/2 in wtPC12 and PC12-27 cells kept for 1hr at rest (0), with NGF (N, 100 ng/ml), rapamycin (R, 1 µM) and the two together (NR). (**C**) The responses induced by the same treatments at the P-Akt(T308). In each cell sample the levels of the ERK (B), Akt and p75^NTR^ (C) proteins did not change during the experiments. The data on P-ERK 1/2(T202/Y204) and P-Akt(T308) are also shown in quantized terms on the right in panels B and C. The numbers flanking the gels are the MDa of the immunolabeled proteins. The statistical analysis and the significance of the differences are shown by the asterisks as specified in the legend for Fig. 1.

In a first series of phosphorylation studies the wtPC12 and PC12-27 cells were incubated in low (1%) serum medium for 24 hr before treatments, and then analyzed in the same medium. The time-course of the TrkA phosphorylation at various tyrosine residues during the first 20 min of NGF treatment is illustrated in Fig. 2A. In the wtPC12, the Y751 site was rapidly phosphorylated, reaching the highest level at 3 min and then declining to the resting level. In PC12-27 cells the Y751 phosphorylation, evident at rest, failed to increase significantly during the stimulation (Fig. 1D,E). The phosphorylation of the Y490 site was well appreciable only in the wt cells, with the highest values at 5–10 min (Fig. 1D,E), whereas the phosphorylation of the Y670, Y674 and Y675, three sites of limited importance for TrkA signaling (Bradshaw et al., 2013) that were investigated together, was similar in the two clones, with only limited changes induced by NGF stimulation (Fig. 1D,E). In conclusion, the level of TrkA appeared similar in the two, low and high REST PC12 clones. In contrast, the NGF-induced autophosphorylation of the receptor, especially that of the Y490 site, was defective. This might be due to the lack of cooperation of the two NGF receptors in the PC12-27 cells.

### The NGF signaling cascades: phosphorylation of ERK and Akt

We next investigated the two major signaling cascades triggered by NGF in PC12 cells, the ERK and the PI3K cascades, analyzed by measuring the specific phosphorylation of the ERK 1/2 and Akt kinase, respectively (Fig. 2). The expression levels of ERK 1/2 were close in the wtPC12 and PC12-27 clones (Fig. 2B), however their NGF-induced phosphorylation at the T202/Y204 sites exhibited different time-courses. In the wtPC12 cells the phosphorylation, low at time 0, reached at 3 min high levels that were maintained for the rest of the experiments (20 min) (Fig. 2A). In PC12-27 cells, the resting level was higher than that of wtPC12. The NGF-induced increase occurred, however it was delayed, reaching levels similar to those of stimulated wtPC12 only after 10 min and thereafter (Fig. 2A).

The investigation was pursued with longer treatments. In these cases, in addition to NGF, we investigated the effects of the classical blocker of the mTORC1 complex, rapamycin, and of the combination NGF/rapamycin. This approach intended to explore the possible role of a negative, mTORC1-induced feed-back that, in the case of insulin and various other growth factors, has been reported to affect the intracellular signaling cascades (Hsu et al., 2011; Huang and Manning, 2009). In neural cells, a mTORC1-induced feed-back had been demonstrated only in relation to a cAMP-induced differentiation (Chin et al., 2010). If the process exists also in relation to the NGF/TrkA system, a blocker of mTORC1 such as rapamycin was expected to block it and thus to increase the NGF-induced response. In contrast, the lack of effect of rapamycin would suggest a lack of the feed-back inhibitory process.

The ERK results obtained by 1 hr treatments with NGF, rapamycin and NGF/rapamycin are shown in Fig. 2B. Upon NGF treatment (Fig. 2B), the levels off ERK 1/2 were unchanged whereas those of their phosphorylation were almost doubled in wtPC12 and significantly increased, although to a moderately lower extent, in PC12-27 cells. In contrast, both the resting and the NGF-induced levels of ERK 1/2, phosphorylation appeared unchanged in both clones upon treatment with rapamycin (Fig. 2B).

Also the levels of Akt were similar in the wtPC12 and PC12-27 cells, with no changes induced by the various treatments with NGF and rapamycin investigated (Fig. 2C). As far as the phosphorylations, that of Akt(T308), indicative of the PI3K cascade, increased slowly in the wtPC12 cells, whereas in the PC12-27 cells it remained apparently unchanged during the first 20 min of NGF treatment (data not shown). One hr (Fig. 2C) or longer (up to 48 hr, data not shown) treatment with NGF induced in wtPC12 cells significant increases of the Akt(T308) phosphorylation. Rapamycin alone, administered for 1 or 24 hr, modified neither the basal nor the NGF-induced Akt(T308) phosphorylation of wtPC12 (Fig. 2C and data not shown). In the PC12-27 cells the basal phosphorylation of Akt(T308) was much lower than that of wtPC12. NGF and, to a lower extent, also rapamycin (administered alone or together for 1 (Fig. 2C) or 24 hr (data not shown)) did apparently induce some increases of P-Akt(T308) which however remained statistically non significant (Fig. 2C).

Two targets of Akt, TSC2(S939) and GSK3β(S9), exhibited phosphorylation patterns different from those of Akt(T308). Specifically, the increases in the wtPC12 cells were smaller while those induced by rapamycin were larger than those of Akt(T308) (compare supplementary material Fig. S1 to Fig. 2C). In the PC12-27 cells, the resting phosphorylation of TSC2(S939) and GSK3β(S9) was low. In these cells no significant increase was induced by NGF. In contrast, rapamycin induced considerable increases (supplementary material Fig. S1). Taken together with the data of Fig. 2C, the data of supplementary material Fig. S1A suggest that some mTORC1-induced feed-back inhibition of the PI3K cascade may operate in PC12-27. In wtPC12 cells, however, no sign of the feed-back was appreciable.

### mTORC1 and mTORC2

Our previous studies had shown mTORC1 to be moderately more active, and mTORC2 much less active in the high REST PC12-27 cells compared to the wtPC12 cells (Tomasoni et al., 2011). In order to investigate the possible involvement in these differences of the NGF receptor signaling and of its mTORC1-dependent feed-back inhibition, we analyzed the direct read-outs of mTORC1 and mTORC2, P-S6(S235/236) and P-Akt(S473), respectively. The latter phosphorylation, considerable only in wtPC12, was completely dissipated by an administration of the PI3K inhibitor, wortmannin, during the last 10 min incubation (supplementary material Fig. S1B). This finding excludes the P-Akt(S473) phosphorylation to be due also to kinases independent of the PI3K cascade, such as IKBE (Guo et al., 2011).

**Fig. 3.**
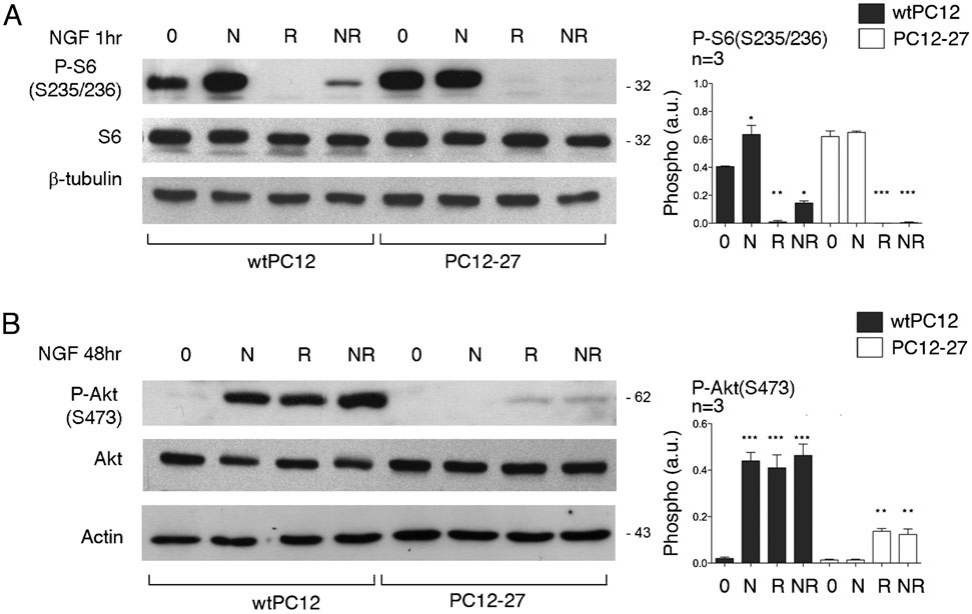
Read-outs of mTORC1 (P-S6(S235/236)) and mTORC2 (P-Akt(S473)) in wtPC12 and PC12-27 cells. (**A**,**B**) wtPC12 and PC12-27 cells were treated for 48 hr with no stimulant (0), with NGF (N, 100 ng/ml), with rapamycin in the last 24 hr (R, 0.1 µM) and with the two together (NR). The quantization of the data is on the right panels. The levels of the S6 and Akt proteins were not changed by the treatments. The numbers flanking the gels are the MDa of the immunolabeled proteins. Statistical analysis and significance of the differences is given as specified in the legend for Fig. 1.

The expression levels of the mTORC1 target, S6, were similar in the two clones. Its basal S235/236 phosphorylation, moderately higher in the PC12-27 cells, was increased upon 1 and 48 hr treatment with NGF, however only in the wtPC12 clone (Fig. 3A and data not shown). The mTORC1 blocker rapamycin, administered alone or together with NGF for 1 to 24 hr, dissipated completely the basal and largely also the NGF-stimulated phosphorylation of the mTORC1 read-out, S6(S235/236), as expected (Fig. 3A and data not shown). We conclude that the moderate differences of mTORC1 activity between wtPC12 and PC12-27 cells, already reported in the two resting clones (Tomasoni et al., 2011), were largely independent of their different NGF signaling.

Fig. 2C had already shown the levels of Akt to be similar in the two clones. These levels were unchanged by 24–72 hr treatment with NGF, 24 hr treatment with rapamycin or by NGF associated to rapamycin during the last 24 hr (Fig. 3B and data not shown). In terms of phosphorylation, the results with the direct mTORC2 read-out, Akt(S473) were quite different in wtPC12 and PC12-27 cells. In the wtPC12, 1 to 24 hr treatment with NGF induced increases of 15–20-fold (Fig. 3B and data not shown). Treatment of wtPC12 with rapamycin induced increases similar to those induced by NGF. The two increases, however, were not additive when the cells were exposed to the combined NGF/rapamycin treatment (Fig. 3B and data not shown). In the PC12-27 clone, the resting phosphorylation of Akt(S473), distinctly lower than that of wtPC12, was not changed significantly by NGF. In contrast rapamycin did increase the P-Akt(S473) by over 5-fold. The combination with NGF did not change the increased P-Akt(S473) induced by rapamycin (Fig. 3B and data not shown).

In order to investigate the possible involvement of the p75^NTR^ receptor in the regulation of the mTORC2 activity we carried out experiments in subclones of wtPC12 cells in which the expression of the receptor had been downregulated by the stable transfection of a specific miRNA. Supplementary material Fig. S2 shows results with a wtPC12 subclone with p75^NTR^ level reduced to approximately 20%, i.e. with a large decrease which however was lower than that of PC12-27 cells, where the receptor is inappreciable. The miRNA transfection did not modify the general phenotype of the wtPC12 cells, that remained largely spherical, different from the flat shape of the PC12-27 cells (Tomasoni et al., 2011). Likewise, the P-Akt(T308) and the P-GSK3β(S9) were unchanged. In contrast, the Akt(S473) phosphorylation was decreased of 35%, suggesting the mTORC2 activity to be reduced (supplementary material Fig. S2). In parallel experiments, the key role of the PI3K cascade in the control of mTORC2 was confirmed by the use of the PI3K inhibitor wortmannin. In both the wtPC12 and PC12-27 clones treated with NGF for 48 hr, treatment with the drug (0.3 µM) for the last 10 min attenuated considerably the P-TSC2(S939) and P-GSK3β(S9) and eliminates completely the P-Akt(S473) phosphorylation (data not shown). Additional subclones, isolated in parallel to the one shown in supplementary material Fig. S2, exhibited lower downregulations of the p75^NTR^ and lower decreases of P-Akt(S473). Summing up, the results confirm that p75^NTR^ has a role in the control of mTORC2 activity, revealed by the direct read-out Akt(S473). The findings with PC12-27 cells strengthen, at the mTORC2 level, the non-significant rapamycin results of the PI3K cascade-dependent phosphorylation of Akt(T308) illustrated in Fig. 2C. In these cells the low mTORC2 activity, unaffected by NGF, appears to be affected by the mTORC1-dependent feed-back inhibition process inhibited by the drug.

### NGF signaling in wtPC12 and PC12-27 cells: role of p75^NTR^

**Fig. 4.**
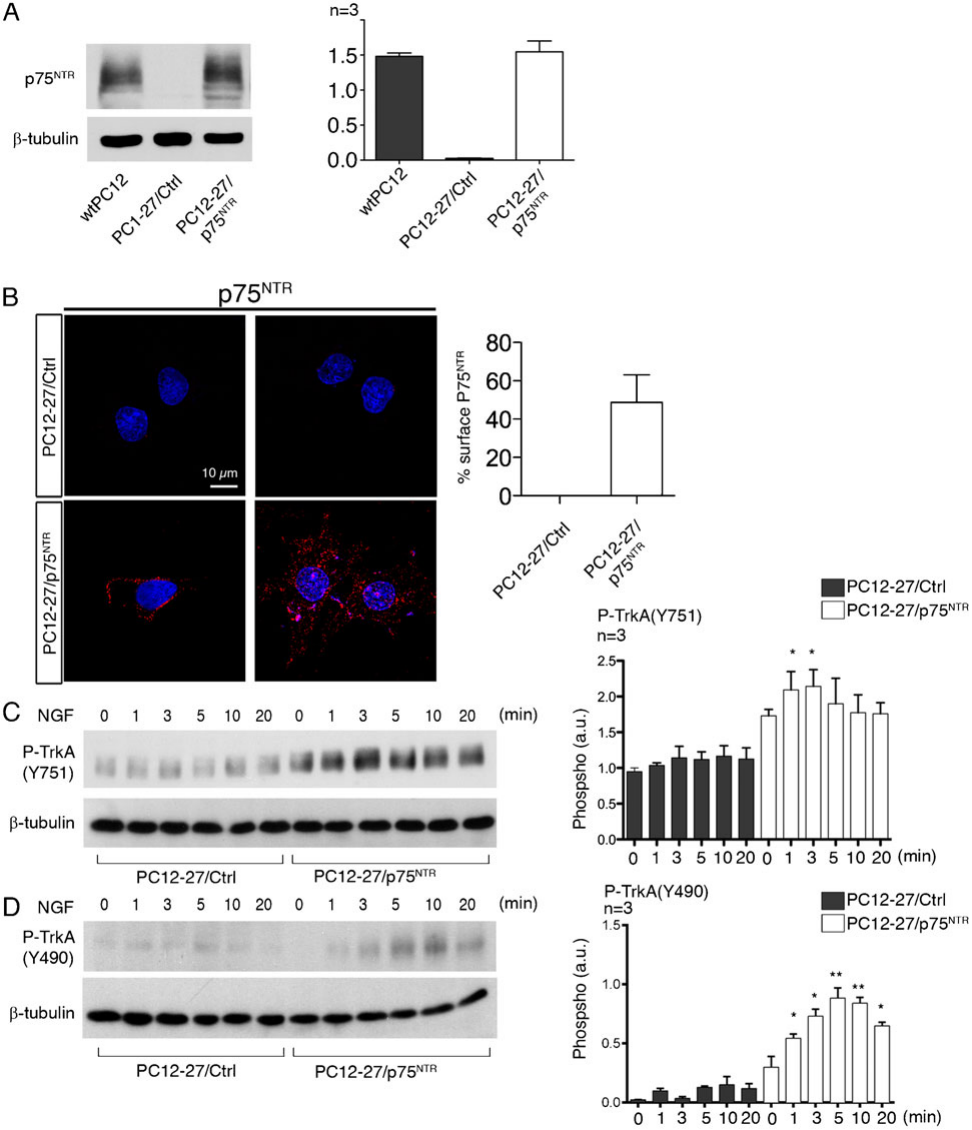
Expression of p75^NTR^ and time-course of TrkA autophosphorylation at the Y751 and Y490 sites in PC12-27 cells transfected with the vector, empty (PC12-27/Ctrl) or including the full length p75^NTR^ (PC12-27/p75^NTR^). (**A**) The western blot of p75^NTR^ in wtPC12, PC12-27/Ctrl and PC12-27/p75^NTR^ cells. Quantization of the data, documenting the similar levels of the receptor in the wtPC12 and PC12-27/p75^NTR^ cells, is on the right. (**B**) The surface immunolocalization of p75^NTR^ in the PC12-27/Ctrl and PC12-27p75^NTR^ cells. The quantization of the results is on the right. Scale bar: 10 µm. (**C**,**D**) The time-course of the TrkA autophosphorylation at the Y751 and Y490 sites induced by NGF (100 ng/ml) in the two transfected subclones, PC12-27/Ctrl and PC12-27/p75^NTR^. The quantization of these data is shown on the right. Statistical analysis and significance of the differences is given as specified in the legend for Fig. 1.

To further investigate the role of p75^NTR^, PC12-27 cells were stably transfected with vectors including the cDNA of the receptor. Among the isolated subclones, one was found to express the receptor at a level and with a surface distribution similar to those observed in the wtPC12 (compare Fig. 4A,B to Fig. 1B,C). To exclude possible artifacts due to hyper/hypo-expression or altered distribution of the receptor, this subclone (labeled PC12-27/p75^NTR^) was selected for subsequent studies, using as control a PC12-27 subclone transfected with the empty vector (PC12-27/Ctrl).

During the first 20 min treatment of PC12-27/p75^NTR^ cells with NGF, the Y751 phosphorylation of TrkA increased markedly, similar to the wtPC12 cells, whereas that PC12-27/Ctrl cells resembled that of the non-transfected PC12-27 cells, i.e. it did not change significantly (compare Fig. 4C to Fig. 1D). At the Y490 site the differences of phosphorylation observed between the cells transfected with and without p75^NTR^ were even larger. In the PC12-27/Ctrl cells this phosphorylation remained almost inappreciable, as in the non-transfected PC12-27 cells, whereas in the PC12-27/p75^NTR^ cells it increased significantly and rapidly upon NGF addition, reaching a maximum at 5–10 min, as in the wtPC12 (compare Fig. 4D to Fig. 1D).

**Fig. 5.**
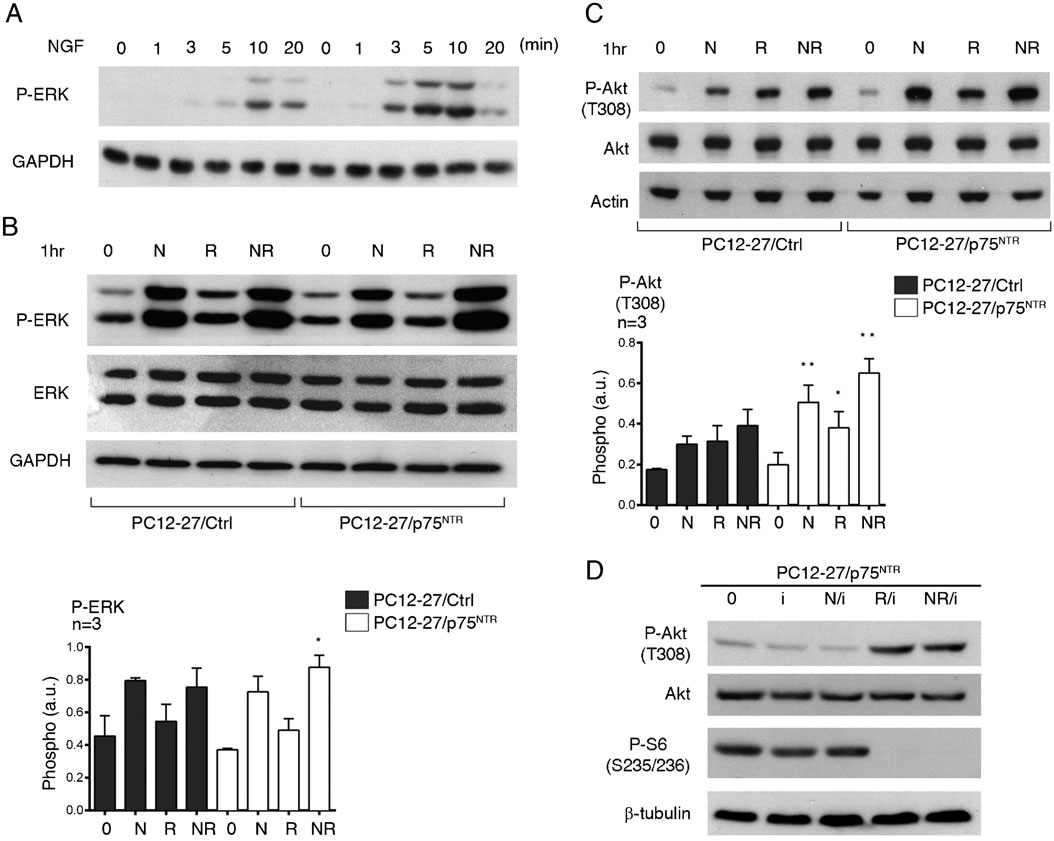
The ERK and PI3K cascades in the PC12-27/Ctrl and PC12-27/p75^NTR^ cells. (**A**) The time-course of ERK phosphorylation, slow in the PC12-27/Ctrl cells, faster in the PC12-27/p75^NTR^ cells. (**B**,**C**) The effects of 1 hr treatment with NGF (N, 100 ng/ml), rapamycin (R, 1 µM) and the two together (NR) on the P-ERK 1/2(T202/Y204) and P-Akt(T308) of the PC12-27/Ctrl and PC12-27/p75^NTR^ cells. The level of p75^NTR^ in the PC12-27/Ctrl and PC12-27/p75^NTR^ cells did not change in response to the treatments. The quantization of the P-ERK 1/2 (T202/Y204) and Akt(T308) phosphorylation data is shown in the panels below the gels. (**D**) The effects of the TrkA receptor inhibitor, Calbiochem 648450 (I, 10 nM, 2 hr), on the P-Akt(T308) and P-S6(S235/236) responses induced by NGF (N, 100 ng/ml), rapamycin (R, 1 µM) administered during the second hr, and the two together (NR). The inhibitor was found to have no appreciable effect on the small increase induced by NGF on the mTORC1 read-out which in contrast was blocked by rapamycin, as expected. In contrast, the inhibitor blocked the effect of NGF (but not that of rapamycin) on P-Akt(308), demonstrating the effect of the neurotrophin on the PI3K cascade to require the cooperation of both p75^NTR^ and TrkA. Statistical analysis of the differences is given as specified in the legend for Fig. 1.

The study of the two cascades, of ERK and PI3K, confirmed the marked changes of the NGF signaling induced in PC12-27 cells by the expression of p75^NTR^. In the case of ERK the phosphorylation of ERK 1/2(T202 and Y204), induced by 1–20 min treatment with NGF, exhibited a faster rate in the PC12-27/p75^NTR^ cells compared to the PC12-27/Ctrl cells (Fig. 5A), similar to the faster rate of the wtPC12 compared to the PC12-27 cells shown in Fig. 2A. Also the similar responses induced in the PC12-27/Ctrl and PC12-27/p75^NTR^ cells by 1 hr treatment with NGF, alone or with rapamycin, resembled the responses induced by the same treatments in the PC12-27 and wtPC12 cells, respectively. With rapamycin alone the changes were small and non significant in both transfected PC12-27 cell subclones (Fig. 5B). In contrast, in the case of P-Akt(T308), the increases in PC12-27/p75^NTR^ cells induced by NGF were larger than in PC12-27/Ctrl (Fig. 5C). Also with the two Akt targets, TSC2(S939) and GSK3β(S9), the phosphorylations induced by NGF and also by rapamycin in the PC12-27/p75^NTR^ were distinctly larger than those in the PC12/Ctrl cells (supplementary material Fig. S3).

A question about the signaling of the PC12-27/p75^NTR^ cells was whether the increases of the ERK and PI3K cascades induced by the expression of p75^NTR^ were dependent on the transfected receptor only or on its cooperation with TrkA. To answer this question we repeated the experiments of Fig. 5B,C by employing PC12-27/p75^NTR^ cells pretreated with a specific inhibitor of the TrkA receptor, Calbiochem 648450 (Yamashita and Tohyama, 2003). Fig. 5D shows that the increased phosphorylation of Akt(T308) induced by NGF was prevented by the pretreatment of the cells with the drug. In order to be generated, the NGF-induced signal requires therefore the two receptors, TrkA and p75^NTR^, to be activated concomitantly. The cooperation with p75^NTR^, however, does not seem to occur only with TrkA. In fact, the responses triggered by rapamycin, alone or together with NGF, were apparently unchanged by the pretreatment of the PC12-27/p75^NTR^ cells with the TrkA inhibitor (Fig. 5D). In PC12 cells the site of action of the mTORC1-dependent feed-back inhibition, the process blocked by rapamycin, is unknown. In other cell types, however, this site has been proposed to coincide with the insulin receptor substrate 1 (IRS1) (Tremblay et al., 2007). This or another post-receptor site may therefore operate in the cooperation of the feed-back inhibition with p75^NTR^. In conclusion, the re-establishment in the PC12-27 of a TrkA/p75^NTR^ ratio analogous to the ratio in wtPC12 was found to rescue the NGF signaling from a partially inactive to a fully active state.

### mTORC1 and mTORC2 in PC12 cells: role of p75^NTR^

**Fig. 6.**
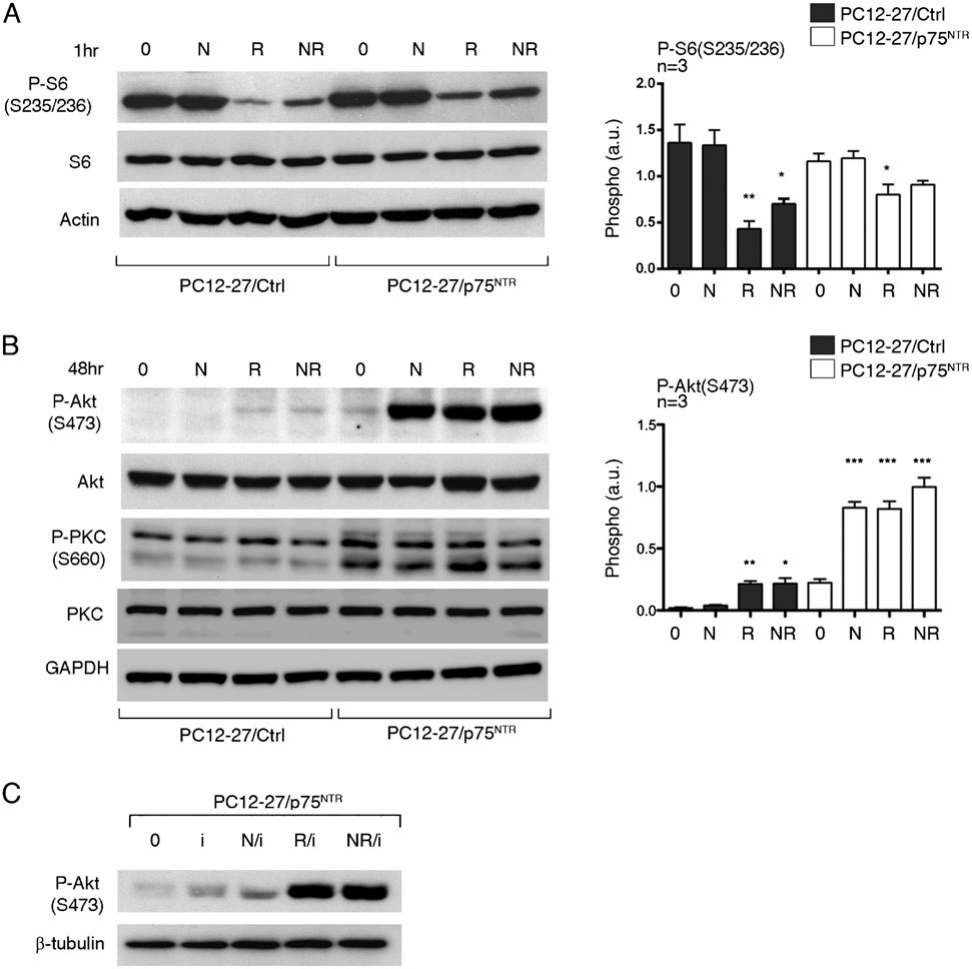
mTORC1 and mTORC2 in the PC12-27/Ctrl and PC12-27/p75^NTR^ cells; effects of the TrkA inhibitor. (**A**) The expression of p75^NTR^ does not change the responses of the mTORC1 read-out, P-S6(S325-326) to 1 hr treatment with NGF (N, 100 ng/ml). Rapamycin (R, 1 µM) induces inhibition of the mTORC1 read-out phosphorylation, which is more extensive in the PC12-27/Ctrl cells. (**B**) The p75^NTR^ transfection induces the rescue of the mTORC2 read-out P-Akt(S473) phosphorylation which is increased markedly by both NGF (N, 100 ng/ml) and rapamycin (R, 1 µM) The phosphorylation of another mTORC2 read-out, PKCα, was high in the PC12-27/p75^NTR^ cells already at rest, with no appreciable changes induced by the treatments. The quantized data of panels A and B in PC12-27/Ctrl and PC12-27/p75^NTR^ cells are given on the right panels. (**C**) Two hr treatment with the TrkA receptor inhibitor, Calbiochem 648450 (I, 10 nM), removed the response triggered in the PC12-27/p75^NTR^ cells by 1 hr treatment with NGF (N, 100 ng/ml), leaving however unchanged that triggered by rapamycin (R, 1 µM), administered alone or combined to NGF. These results demonstrate 1) that the NGF response is mediated by the cooperation of the TrkA and p75^NTR^ receptors; and 2) that the mTORC1.induced feed-back block by rapamycin cooperates with p75^NTR^ working however not at the TrkA receptor but at a post-receptor site. Statistical analysis of the differences on the right in panels A and B is given as specified in the legend for Fig. 1.

The lack of p75^NTR^ could be the cause of the differential activity of mTORC1 and mTORC2 in PC12-27 with respect to wtPC12 cells. Fig. 6A shows that, with mTORC1, the change of activity induced by the stable expression of p75^NTR^ was minor. With respect to the PC12-27/Ctrl cells the phosphorylation of the direct (S235/236) S6 read-out was in fact only moderately lower in the resting and NGF-treated PC12-27/p75^NTR^ cells, and the inhibitory effect of rapamycin was also lower (Fig. 6A). The situation was profoundly different with mTORC2 (Fig. 6B). In the PC12-27/Ctrl cells the read-out P-Akt(S473) was similar to the non-transfected PC12-27 cells, i.e. the read-out was unchanged by NGF and increased only little after treatment with rapamycin, (compare Fig. 6B to Fig. 3B). In contrast, in the PC12-27/p75^NTR^ cells the phosphorylation of the read-out after NGF, rapamycin and the two together was much stronger, approaching values similar to those observed in the wtPC12 (compare Fig. 6B to Fig. 3B). Fig. 6B shows also that, in the PC12-27/p75^NTR^ cells, the phosphorylation of another read-out of mTORC2, PKCα (Guertin et al., 2006) was much higher than that of the PC12-27/Ctrl already at rest, confirming the dependence of mTORC2 on the p75^NTR^ expression. In this case the treatments with NGF and rapamycin induced only marginal effects. Moreover, similar to the results of P-(T308)Akt (Fig. 5D), also the responses of the mTORC2 read-out P-(S473)Akt induced in the PC12-27/p75^NTR^ cells by the treatment with NGF were inhibited by the specific TrkA blocker drug, Calbiochem 648450 (Yamashita and Tohyama, 2003). In contrast, the responses to rapamycin, acting by removal of the mTORC1 feed-back inhibition, were not (Fig. 6C). Together with the data of Fig. 5D these results confirm the cooperation between the TrkA and the p75^NTR^ signaling to be necessary for the NGF-induced mTORC2 activation. In contrast the cooperation appears unnecessary when the signal is triggered not at the level of TrkA but (by rapamycin) at a post-receptor site. Summing up, the expression of p75^NTR^ in the PC12-27 cells appears to modify both the signaling of NGF and the activity of mTORC2, bringing them to levels approaching those of wtPC12.

### About the phenotype of PC12-27/p75^NTR^ cells

**Fig. 7.**
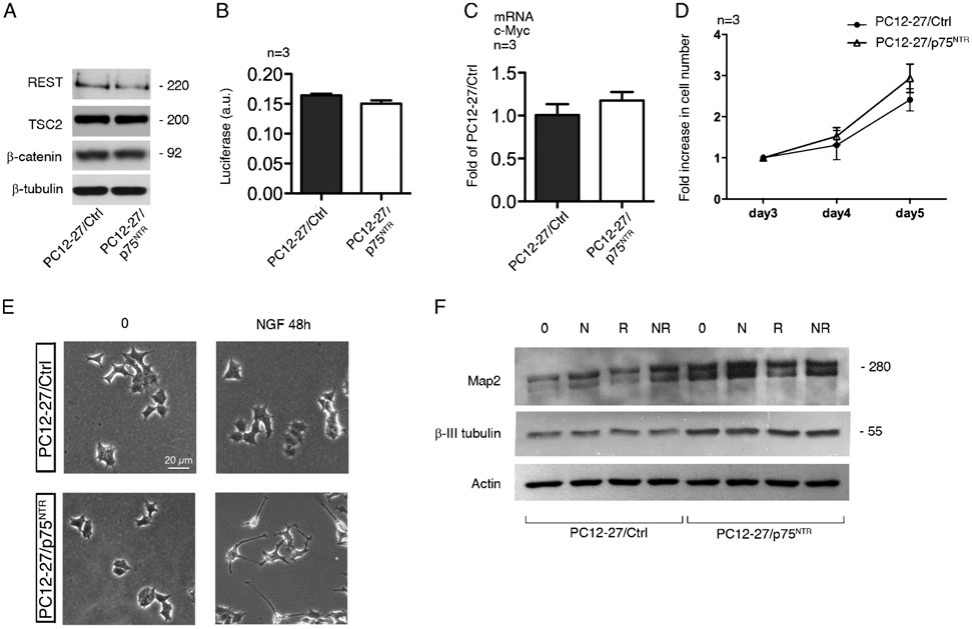
Phenotype of the PC12-27/Ctrl and PC12-27/p75^NTR^ cells. (**A–D**) No difference exists between PC12-27/Ctrl and PC12-27/p75^NTR^ in a number of important features: the levels of the REST, TSC2 and β-catenin proteins (A); the β-catenin-dependent transcription revealed by a luciferase assay (B); the expression of the β-catenin-target gene, cMyc (C); the rate of cell proliferation (D). (**E**) Phase contrast images of the PC12-27/Ctrl and PC12-27/p75^NTR^ before and after a 48 hr treatment with NGF (100 ng/ml). Scale bar: 20 µm. (**F**) The expression of two neuronal markers, Map2 and β-III tubulin, in PC12-27/Ctrl and PC12-27/p75^NTR^ cells incubated for 48 hr with no treatment, with NGF (N, 100 ng/ml), rapamycin (R, 0.1 µM during the last 24 hr) and the two together.

We then investigated in PC12-27/p75^NTR^ cells a few properties known to distinguish the PC12-27 from wtPC12 cells, i.e. the very high REST (D'Alessandro et al., 2008), the low TSC2 and the high β-catenin (Tomasoni et al., 2011). The question was whether these properties also depend on the lack of the p75^NTR^ receptor. Fig. 7A shows that this is not the case. In fact the levels of the three factors in the PC12-27/p75^NTR^ cells were as high as in the PC12-27/Ctrl cells. Also a few functional properties dependent on the high β-catenin, i.e. the luciferase assay of its transcription activity, the expression of a transcription target, the cMyc oncogene, and the high cell proliferation (Tomasoni et al., 2011), were not changed significantly by the p75^NTR^ transfection (Fig. 7B–D).

Finally, we investigated whether, and to what extent, the expression of p75^NTR^ modifies two aspects of the phenotype sensitive to NGF that are greatly defective in PC12-27 cells, the outgrowth of neurites and the expression of neuron-type markers. Fig. 7E compares the morphology of the PC12-27/Ctrl and PC12-27/p75^NTR^ cells, at rest and upon 48 hr treatment with NGF. The flat structure of PC12-27/Ctrl cells, similar to that of the non-transfected PC12-27 cells (Tomasoni et al., 2011) was hardly affected by the 48 hr treatment with NGF. In the PC12-27/p75^NTR^ cells, on the other hand, the shape was not changed much, however the NGF-induced neurite outgrowth response was evident in terms of both number of neurites sprouted per cell and average neurite length (Fig. 7E). In the resting PC12-27/Ctrl cells the levels of the two neuronal markers investigated, Map2 and β-III tubulin, were low. Treatment for 48 hr with NGF, 24 hr with rapamycin or the two together induced only small or no increases. In PC12-27/p75^NTR^ cells, the resting levels of the two markers were higher, however the increases induced by NGF and rapamycin were small and non significant (Fig. 7F).

## Discussion

The focus of this study was the cooperation of the two specific receptors of NGF, TrkA and p75^NTR^. The parallel investigation of two clones of PC12, wtPC12 and PC12-27, was a good model for these studies. In fact, the wtPC12 cells exhibit the well-known properties of the cell line, including the expression of both NGF receptors. In contrast the PC12-27 cells, due to their very high level of the transcription repressor REST, lack the p75^NTR^ receptor. In our previous studies, the PC12-27 cells were reported to be defective also in TrkA, based however only on immunocytochemical results obtained with a single anti-TrkA antibody (Schulte et al., 2010). Further studies by RT-PCR and western blotting, and the use of anti-TrkA antibodies from other sources, have demonstrated now that in PC12-27 cells both the expression and the surface distribution of TrkA are similar to those of wtPC12 cells. By stable transfection of p75^NTR^ we were able to recreate, in PC12-27 cells, a TrkA/p75^NTR^ ratio and a p75^NTR^ surface distribution analogous to those of the wtPC12 cells. Moreover, the direct comparison of PC12-27 cells transfected with or without the p75^NTR^ provided us with a direct picture of the changes introduced in a neural cell by the rescue of the receptor. Such a picture reveals new aspects because, even if p75^NTR^ null mice had been available for many years, the cellular studies of NGF signaling in neural cells in which p75^NTR^ had been blocked or downregulated were still a few (Bui et al., 2002; Ceni et al., 2010).

The differential properties of the PC12-27 cells with respect to wtPC12 are known in detail. Several of such properties, such as the lack of neurosecretion, the marginal NGF-induced neurite outgrowth and the high rate of proliferation were i) introduced in wtPC12 by the increase of their REST level and ii) attenuated or removed from PC12-27 by the decrease of their high REST activity (D'Alessandro et al., 2008; Schulte et al., 2010; Tomasoni et al., 2011). Moreover, the differential properties of PC12-27 are not peculiar of this clone, but are shared by other neural cells, such as the SH-SY5Y and NT2/D1 lines, and even embryonal rat cortex neurons and astrocytes, in which high REST is expressed spontaneously or induced by transfection (D'Alessandro et al., 2008; Racchetti et al., 2010; Tomasoni et al., 2011). Finally it should be emphasized that, in all cells, the level of REST is not fixed but can increase in various physiological and pathological conditions (Calderone et al., 2003; Guardavaccaro et al., 2008). The results now obtained with PC12-27 cells, therefore, may be relevant also to other neural cells when exhibiting high REST levels.

So far, several studies on the role of NGF in PC12-27 cells were focused not on the mechanisms but only on the final effects of the neurotrophin, in particular neurite outgrowth and the expression of neuronal markers (Leoni et al., 1999; Schulte et al., 2010). Here we have extended the study to TrkA autophosphorylation and to its two main intracellular cascades, the ERK and PI3K cascades. At the TrkA receptor the lack of p75^NTR^ was found to affect the NGF-induced phosphorylation primarily at one tyrosine, Y490, the initiation site of the PI3K cascade (Bradshaw et al., 2013; Kaplan and Miller, 2000). Consistently with this finding the P-(T202/Y204)ERK, the marker of the ERK cascade, reached levels analogous to those of the wtPC12, although with some delay. In contrast the P-Akt(T308), the marker of the PI3K cascade, remained inappreciable for quite some time and very low even after hours of treatment (Ito et al., 2003). Interestingly, the P-(T202/Y204)ERK of PC12-27 cells was unaffected by the mTORC1 blocker rapamycin, whereas the P-Akt(T308) was stimulated, suggesting the PI3K cascade of PC12-27 cells to operate under the negative feed-back control of mTORC1. In this respect it is interesting that, in cultured neurons IRS1, the proposed target of the feed-back (Tremblay et al., 2007) had been reported to operate in association with PI3K (Miranda et al., 2001; Yamada et al., 1997). Taken together, and at variance with the previous conclusions of Leoni et al. and Schulte et al. (Leoni et al., 1999; Schulte et al., 2010), our results demonstrate that the NGF signaling of the p75^NTR^-defective PC12-27 cells is not completely, by only partially inactive. Specifically, in these cells the NGF-induced autophosphorylation of TrkA at Y490 appears greatly reduced, the activation of the ERK cascade is largely maintained, whereas that of the PI3K cascade is severely affected.

To investigate the consequences of the p75^NTR^ rescue we did investigate a stably transfected PC12-27 subclone in which the ratio of the receptor to TrkA and its distribution at the cell surface were similar to those of wtPC12. This choice minimized the risk of artifacts due to defects in the interaction of the two receptors. Among the isolated subclones, however, only one, labeled PC12-27/p75^NTR^, exhibited these properties, and only this subclone was investigated. Therefore our present data remain to be confirmed in other subclones. Nevertheless, the results obtained appear promising because a number of the NGF-induced properties typical of wtPC12, i.e. the active PI3K cascade, neurite outgrowth and the acquisition of neuronal markers, were rescued in the PC12-27 by the transfected p75^NTR^ working in cooperation with TrkA. Other differential properties of the PC12-27 clone, however, were not modified by the transfection of p75^NTR^, suggesting their independence on the NGF signaling. These properties include the high level of REST, which is spontaneous in the clone; the low level of TSC2; the high level and the transcription activity of β-catenin. High REST, low TSC2 and high β-catenin are the three coordinate factors that account for the high proliferation rate of PC12-27 cells (Tomasoni et al., 2011), a property that was also unaffected by the transfection of p75^NTR^.

Our results provided some evidence about the links between NGF signaling and the mTORCs. The stimulation of the PI3K cascade by rapamycin, visible however only in PC12-27 cells, concurs with the previous data of Chin et al. to suggest the existence of an mTORC1-dependent feed-back inhibition of signaling also in neural cells (Chin et al., 2010). The data on mTORC2 were obtained by studying its direct read-outs, P-Akt(S473) and PKCα. The transfection of p75^NTR^ was found to induce marked increases of the mTORC2 activity. This effect was not a consequence of p75^NTR^ only but required the cooperation of TrkA, as shown by its disappearance induced by the specific inhibitor of that receptor, Calbiochem 648450. The activity of mTORC2, critical for both neural and non neural cells (Oh and Jacinto, 2011), takes place under the control of two main mechanisms, dependent one on the PI3K cascade, the other on the TSC complex (Dalle Pezze et al., 2012; Guertin et al., 2006; Huang et al., 2008; Shanmugasundaram et al., 2013). In the non-transfected PC12-27 cells the TSC complex is little active due to the reduced level of TSC2 (Tomasoni et al., 2011). The TSC complex could therefore have a role in the low activity of mTORC2. However, the TSC2 level did not increase in the PC12-27/p75^NTR^ cells. Therefore the mTORC2 rescue observed in the latter cells might be due mostly to the PI3K cascade which is also rescued by the transfection. These data are new. Because of their relevance they deserve to be further investigated by additional approaches not yet employed, including the downregulation of specific components of the mTORC2 complex such as Rictor.

mTORC2 is known to play important roles in many, and possibly all types of cells. In non-neural cells, and especially in their tumors, it can contribute to proliferation, migration, invasion, metastases and other functions (Kim et al., 2011; Shanmugasundaram et al., 2013; for a review, see Oh and Jacinto, 2011). In some non-neural cells and tumors, such as myoblasts, lymphocytes B and T and leukemias, however, mTORC2 has been shown to induce primarily differentiation (Lazorchak and Su, 2011; Lee et al., 2012; Matheny et al., 2012; Shu and Houghton, 2009). In the case of neural cells an mTORC2-dependent differentiation, induced via the inhibition of FoxO3a (Wang et al., 2013), is the only effect reported so far (Carson et al., 2013; Dimitroff et al., 2012; Urbanska et al., 2012). Our results seem to add a new example along this line.

**Fig. 8.**
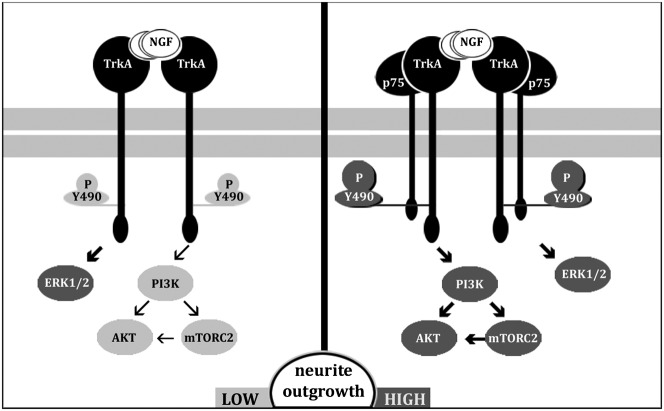
Model of the changes of the NGF signaling induced in PC12-27 cells by the transfection of p75^NTR^. The panel to the left illustrates the signaling in the non-transfected PC12-27 cells. Activation of TrkA by NGF binding induces a weak autophosphorylation at the Y490 site which is followed by a dissociation of the two signaling cascades, indicated by both the thickness of the arrows and the tone of the kinases: the ERK1/2 cascade is strong whereas the PI3K cascade is weak. The panel to the right shows that activation by NGF of the TrkA/p75^NTR^ complex induced a stronger autophosphorylation of Y490 accompanied by the parallel strong activation of both the cascades. In the first case the neurite outgrowth response remains low; in the second it is strong.

In conclusion, the comparison of the canonical wtPC12 with PC12-27, a clone characterized by high REST and by the ensuing lack of the p75^NTR^ receptor, has revealed new aspects of NGF signaling. wtPC12 and PC12-27 were found to differ from each other for many properties. PC12-27 cells exhibited, on the one hand, the mTORC1-dependent feed-back control process (that was inappreciable in the wt clone); on the other hand, a strong defect of the PI3K cascade, including a low activity of mTORC2. The rescue of p75^NTR^, with the reestablishment of its cooperation with TrkA, largely normalized the PI3K/mTORC2 signaling (see the model in Fig. 8) as well as neurite outgrowth. The properties normalized in the transfected PC12-27 appear therefore to depend on the p75^NTR^/TrkA signaling. This mechanism appears in contrast to those of other properties (high levels of REST and β-catenin; low level of TSC2; low rate of proliferation), also exhibited by PC12-27 cells, that were unaffected by the rescue of p75^NTR^.

## Materials and Methods

### Antibodies and chemicals

The anti-p75^NTR^ extracellular domain (REX) rat polyclonal antibody (pAb) was a gift of L. Reichardt. The other antibodies were from the following commercial sources: anti-P-Akt(S473), anti-P-Akt(T308), anti-GSK3β, anti-P-GSK3β(S9), anti-TSC2, anti-PKCα(S660), and anti-S6 monoclonal antibodies (mAbs); anti-TrkA, anti-P-TrkA(Y490), anti-ERK 1/2 and anti-P-ERK 1/2(T202/Y204), anti-P-TSC2(S939), anti-Akt and anti-P-S6(S235/236) rabbit pAbs, Cell Signaling; anti-P-TrkA(Y751) and anti-P-TrkA(Y670/Y674/Y675) rabbit pAb: Invitrogen; anti-TrkA C20 extracellular domain rabbit pAb, anti-actin goat pAb: Santa Cruz; anti-p75^NTR^ rabbit pAb, Promega; anti-REST rabbit pAb: Upstate; anti-β-tubulin mAb and anti-actin rabbit pAb: Sigma; anti-β-catenin mAb: BD Transduction; anti-Map2 mAb, Millipore; anti-β-III tubulin mAb, Covance; FITC-conjugated and TRITC-conjugated goat anti-rabbit pAbs, and goat anti-mouse IgG subclasses: Southern Biotech.; horseradish peroxidase-conjugated goat anti-mouse and anti-rabbit pAbs: Bio-Rad. NGF was from Alomone; the BCA Protein Assay Kit from Pierce; rapamycin and the TrkA inhibitor 648450 from Calbiochem; the fluorescent DNA-binding probe DAPI, wortmannin and other chemicals, from Sigma–Aldrich.

### Cell clones

The PC12 clones, wtPC12 and PC12-27, were as described (D'Alessandro et al., 2008). Subclones were generated in this work. Cells of clones and subclones were grown in DMEM medium supplemented with either 1% (starvation) or 10% horse serum, and incubated at 37°C.

### Transfections

Stable transfections were as described (D'Alessandro et al., 2008) using lipofectamine 2000TM (Invitrogen). PC12-27 cells were co-transfected with the pcDNA3.1Hygro(+) together with the plenty-OE-sRFP vector including the full length human p75^NTR^ cDNA, to generate cells with stable p75^NTR^ expression; wtPC12 cells received the pRRLsinPPT vector with the p75^NTR^ miRNA, to downregulate the receptor. Both vectors were gifts of P.A. Barker (Ceni et al., 2010). The controls received the vectors only. The transfections were confirmed by qPCR and immunoblotting. For the luciferase assay, nt5iD90βcat/GFP construct (gift of A. Chenn and C.A. Walsh) or the vector backbone pEGFP-1 were used for the stable transfection of PC12-27/Ctrl and PC12-27/p75^NTR^ cells. Subclones were grown in complete medium supplemented with 500 µg/ml of G418.

### Cell proliferation

To investigate cell proliferation, the clones were plated at 1×10^4^/well in a 24 well dish. Medium was replaced every 48 h. Upon 3, 4 and 5 days in culture the cells were trypsinized and counted after Trypan Blue exclusion.

### q-PCR

Total RNA was extracted using RNeasy mini columns (Qiagen), following manufacturer's instructions, and its concentration was determined by spectrophotometry. 1–2 µg of total RNA were used to generate cDNA templates for RT-PCR, using the RevertAid First Strand cDNA Synthesis kit in accordance with the manufacturer's protocol (from Thermo Scientific). Real-time PCR was performed on a LightCycler® 480 System (FastStart DNA Master SYBR Green I of Roche Appl. Sci.) according to a standard protocol, using 50 ng template cDNA. All primers were used at the final concentration of 500 nM. Values were normalized to the concentration of calmodulin mRNA. Values are expressed as either fold of wt or PC12-27/Ctrl cells.

### Western blotting

Total cell extracts were obtained by suspending cells in lysis buffer containing 1% Triton X-100, 50 mM Tris-HCl, pH 7.5, 250 mM NaCl, 5 mM EDTA, 50 mM NaF, together with protease and phosphatase inhibitors (Xu et al., 2003). To investigate the time-course of TrkA phosphorylation the cells were transferred rapidly on ice and then suspended in lysis buffer containing 10 mM Tris-HCl pH 7.8, 150 mM NaCl, 1 mM EDTA, 1% (v/v) Nonidet P40, 1% (w/v) sodium deoxycholate, with protease and phosphatase inhibitors. The lysates were cleared by centrifugation at 16.000 *g* for 20 min at 4°C, and the supernatants were analyzed (Takahashi et al., 2011). Proteins were quantified by BCA assay and appropriate amounts (most often 30 µg) were separated by SDS-PAGE. After transfer to nitrocellulose filters, they were immunolabeled as described (Dignam et al., 1983). Photographic development was by chemiluminescence (ECL, Amersham Bioscience or Immobilon substrate, Millipore). Western blot bands were quantified by the ImageJ program (rsb.info.nih.gov/ij), normalized to markers that do not change their concentration during the experiment (β-tubulin, actin or GAPDH) immunolabeled in parallel. Data are expressed as arbitrary units (a.u.).

### Luciferase assay

β-Catenin transcription assay was performed using the Dual-Luciferase reporter assay kit (Promega). The 16× TOPFLASH reporter plasmid (1 µg) (gift of R.T. Moon and H.H. Das Gupta) and 100 ng of SV40-Renilla-luc were cotransfected using lipofectamine 2000TM, and luciferase activity was measured 24 hr later, using a luminometer (GloMax Multi Detectionm System of Promega). Data are expressed as a.u.

### Immunofluorescence and bright field microscopy

The immunofluorescence experiments were performed as described (Tomasoni et al., 2011). Specifically, cell monolayers on coverslips were fixed with 4% formaldehyde for 10 min at room temperature and quenched in 0.1 M glycine, then processed directly or permeabilized for 20 min in PBS containing 0.2% Triton X-100 and 1% bovine serum albumin, and finally immunolabeled for 1 hr with either anti-TrkA or anti-p75^NTR^ pAbs, the latter against the whole receptor molecule (C14 and Promega) or against its extracellular domain (C20 and REX), diluted in PBS with 1% BSA. The bound antibodies were stained with FITC-conjugated and TRITC-conjugated goat anti-rabbit pAbs, or goat anti-mouse IgG subclasses. In some cases nuclei were stained with DAPI. Samples were studied in a PerkinElmer Ultraview ERS confocal microscope. Image deconvolution was performed in a wide field microscope of the Delta Vision system.

### Statistical analyses

The significance of the data was assessed using the two-tailed unpaired t-test and the Anova test, making reference to the unstimulated samples of both the controls and the variously stimulated cell preparations. Data shown are means ± s.e. The number of experiments is specified in the figures or figure legends. *P*<0.05 is considered significantly different. In the figures, ***P<0.001; **P<0.01; *P<0.05.

## Supplementary Material

Supplementary Material
