# Supplementary material for: NGF signaling in PC12 cells: the cooperation of p75NTR with TrkA is needed for the activation of both mTORC2 and the PI3K signalling cascade
Source: Biol Open. 2013 Jul 12;2(8):855–66. doi: 10.1242/bio.20135116 (PMC3744078; doi:10.1242/bio.20135116)
Supplement: Supplementary Material [file supp_2_8_855__index.html]

NGF signaling in PC12 cells: the cooperation of p75NTR with TrkA is needed for the activation of both mTORC2 and the PI3K signalling cascade — NGF signaling in PC12 cells: the cooperation of p75NTR with TrkA is needed for the activation of both mTORC2 and the PI3K signalling cascade — Supplementary Material 

# NGF signaling in PC12 cells: the cooperation of p75NTR with TrkA is needed for the activation of both mTORC2 and the PI3K signalling cascade

## 

**Files in this Data Supplement:**

- Supplementary Material - Sara Negrini et al. doi: 10.1242/bio.20135116
