# Supplementary material for: NGF signaling in PC12 cells: the cooperation of p75NTR with TrkA is needed for the activation of both mTORC2 and the PI3K signalling cascade
Source: Biol Open. 2013 Jul 12;2(8):855–66. doi: 10.1242/bio.20135116 (PMC3744078; doi:10.1242/bio.20135116)
Supplement: Supplementary Material [file supp_bio.20135116_bio.20135116-s1.pdf]

## Supplementary Material

**Sara Negrini et al. doi: 10.1242/bio.20135116**

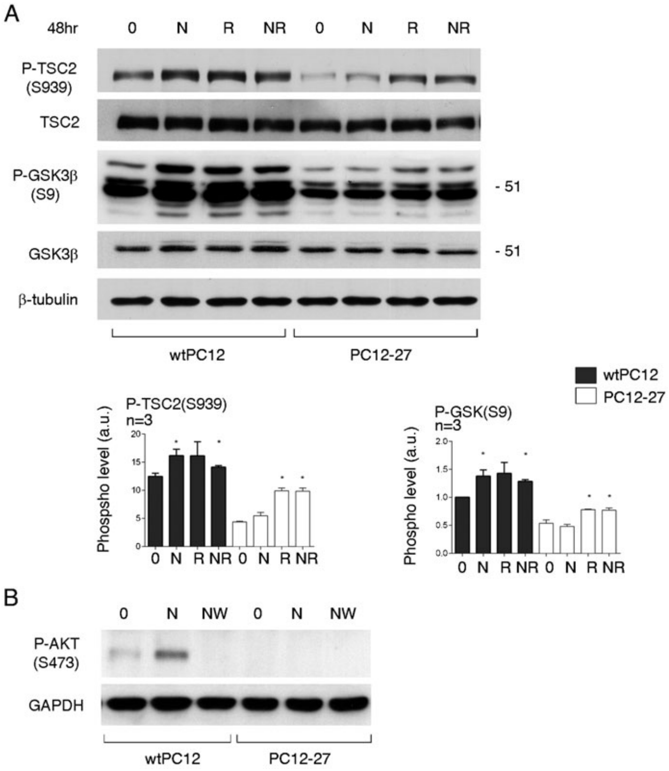


**Fig. S1. Phosphorylation of two Akt targets, TSC2(S939) and GSK3β(S9), induced by NGF and rapamycin in wtPC12 and PC12-27 cells.** (**A**) The changes in phosphorylation of TSC2(S939) and GSK3β(S9) induced in the two cell clones by 1 hr treatment with NGF (N, 100 ng/ml), rapamycin (R, 1 μM) or the two together. The numbers flanking the gels are the MDa of the immunolabeled proteins. Compared to the results of P-Akt(T308), shown in Fig. 2C, the changes of the two targets induced by rapamycin are larger, especially in the PC12-27 cells that are unresponsive to NGF. (**B**) The phosphorylation of Akt(S473) induced by NGF (N, 100 ng/ml, 60 min) in wtPC12 cells was completely dissipated by the addition of wortmannin (NW, 0.3 μM) during the last 10 min.

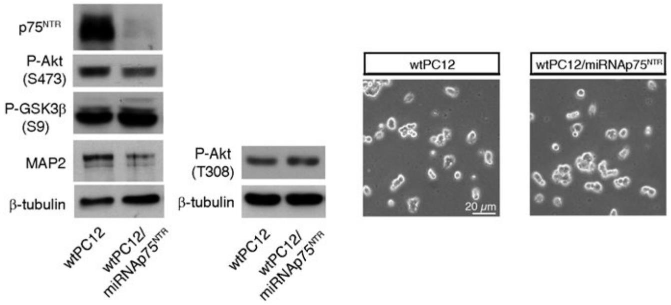


**Fig. S2. Changes of wtPC12 induced by the transfection of a p75NTR miRNA.** In the subclone illustrated here, in which the level of the receptor was decreased of ~80%, the decrease of the mTORC2 read-out p-Akt(S473) was decreased of ~35%. These changes were apparently ineffective on the PI3K cascade since the P-Akt(T308) and P-GSK3β(S9) were unchanged, while the level of the neuronal marker Map2 was significantly decreased. The general phenotype of the wtPC12 cells was apparently unaffected by the miRNA expression. Scale bar: 20 μm.

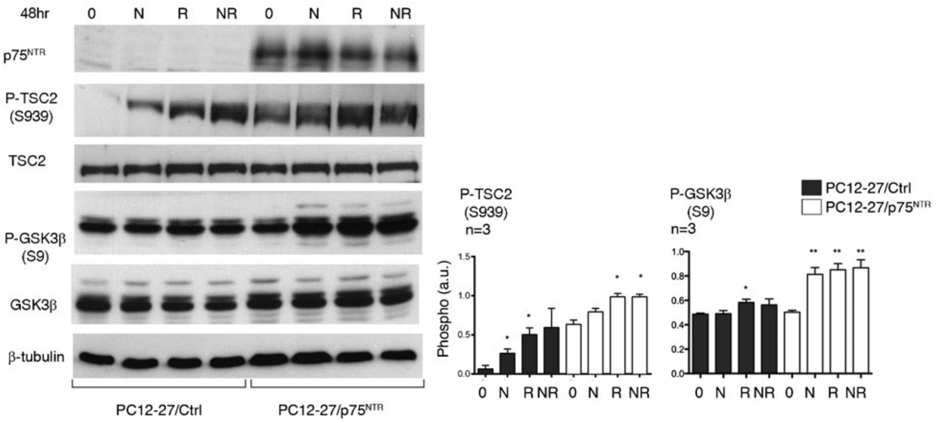


**Fig. S3. Phosphorylation of two Akt targets, TSC2(S939) and GSK3β(S9), induced by NGF and rapamycin administered for 48 and 24 hrs, respectively, in PC12-27/Ctrl and PC12-27/p75NTR cells.** The results in the PC12-27/Ctrl cells resemble those obtained with the PC12-27 cells illustrated in supplementary material Fig. S1. The transfection of p75NTR induces an increased phosphorylation that in the case of GSK3β(S9) occurs not only upon treatment with rapamycin (R, 1 μM) but also with NGF (N, 100 ng/ml).
